# Supplementary material for: CiXTH29 and CiLEA4 Role in Water Stress Tolerance in Cichorium intybus Varieties
Source: Biology (Basel). 2023 Mar 13;12(3):444. doi: 10.3390/biology12030444 (PMC10045840; doi:10.3390/biology12030444)
Supplement: Supplementary file 1 [file biology-12-00444-s001.zip › biology-2223506-supplementary.pdf]

Table S1. Primer used for cloning and RT-qPCR analysis.

Primer Name	Sequence	Analysis
*Ci*Act2for	5’-CCAAATCCAGCTCATCAGTCG-3’	RT-qPCR
*Ci*Act2rev	5’-TCTTTCGGCTCCGATGGRGAT-3’	RT-qPCR
*Ci*Tub2for	5’-GCACGGCATTGATGTGACC-3’	RT-qPCR
*Ci*Tub2rev	5’-GAACAACCTCCCGCCACT-3’	RT-qPCR
*At*XTH29-3’clonefor	5’-TGGTATCGTCGTCGCTTTCT-3’	Cloning
*At*XTH29-3’clonerev	5’- GTCCAGTATCTCTAAACCGG-3’	Cloning
*Ci*XTH29RTfor	5’-TGATAAAGTTGCCAGGAGCG-3’	RT-qPCR
*Ci*XTH29RTrev	5’-AGGCTTTCCTTCTAGGTTCC-3’	RT-qPCR
*Cs*LEA4for	5’-CGGCTTGGGTATGCTAATGT-3’	RT-qPCR
*Cs*LEA4rev	5’- CGTCAAGGCAAGAAAAATTG -3’	RT-qPCR

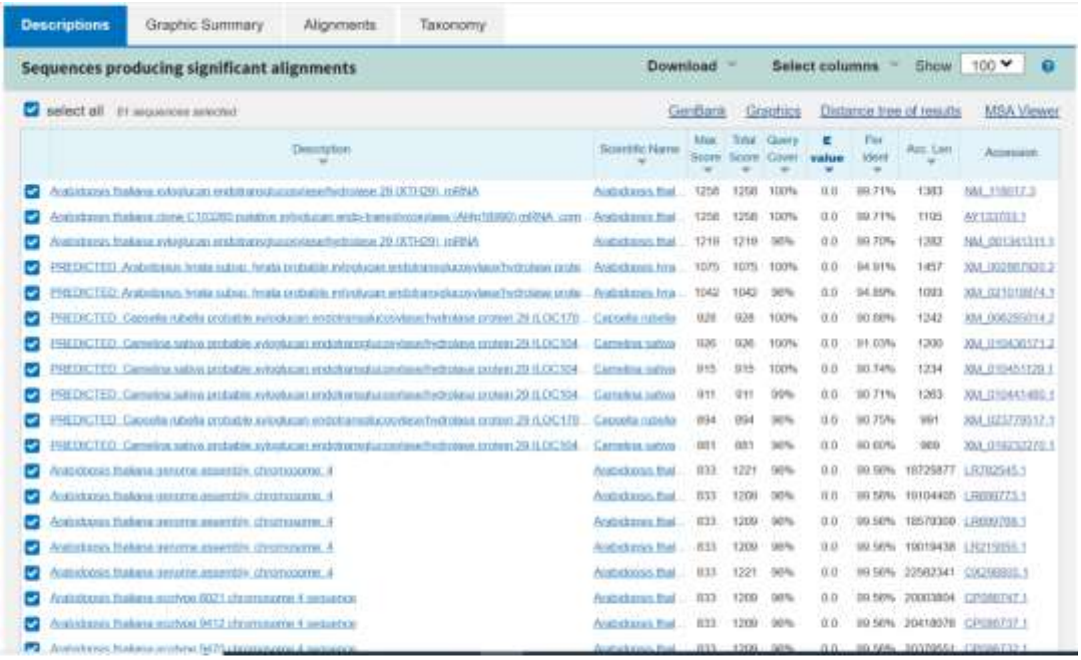

A)

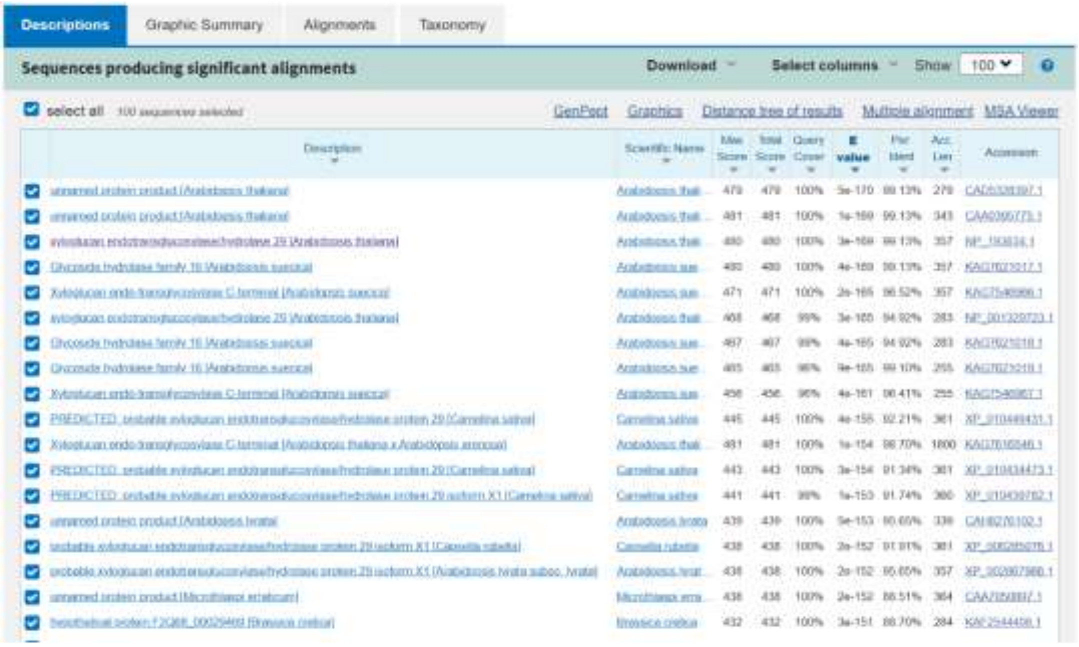

B)

Figure S1. Comparison of *CiXTH29* nucleotide (A) and amino acid (B) sequence with all the other sequences present in BLAST database.

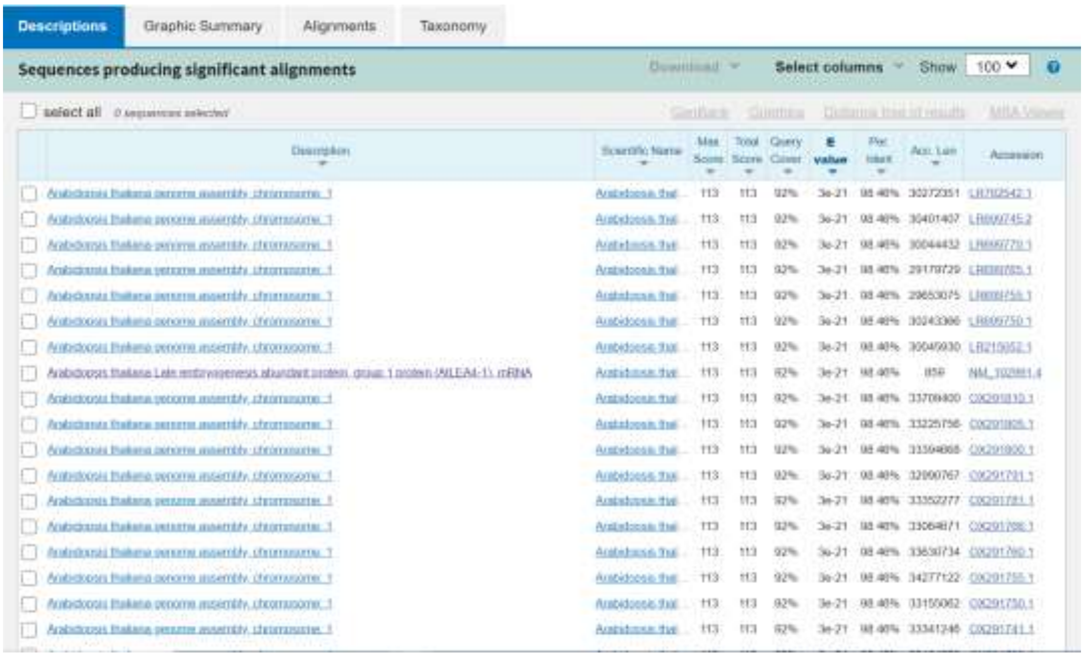

A)

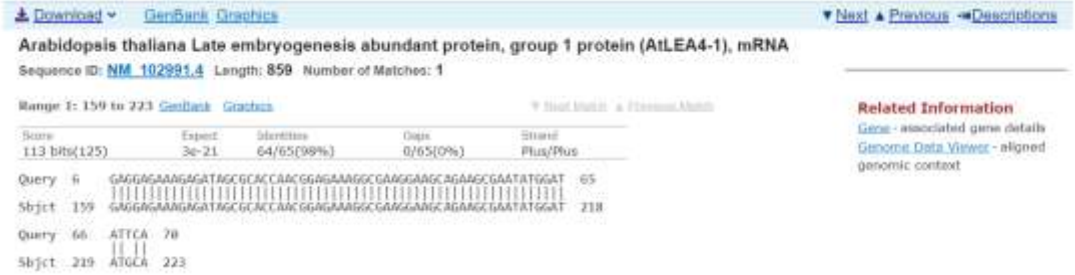

B)

Figure S2. Comparison of *CiLEA4* nucleotide sequence with all the other sequences present in BLAST database (A). Alignment of *CiLEA4* PCR product (query) with *AtLEA4* (Sbjct) (B).

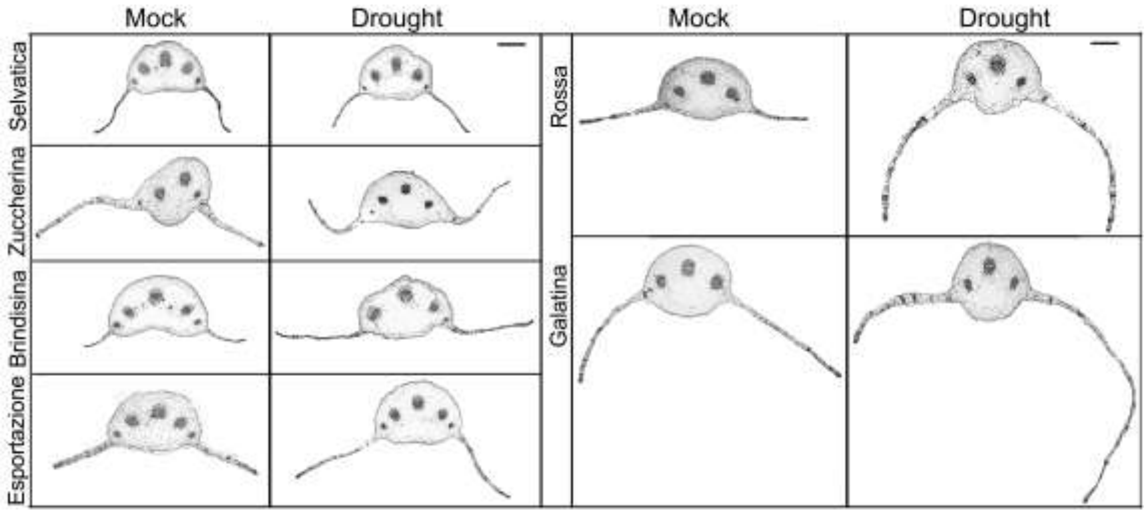

Figure S3. Bright field images of leaf lamina thin transverse section of 6-week-old chicory varieties in mock and drought stress conditions.

Scale bar = 1 mm.

Table S2. Validation of reference genes in *C. intybus*. Ct values of the candidate reference genes *CiAct2* and *CiTub2* in 6-week-old chicory plants kept for further 10 days with (Mock) or without watering (Drought). Ct values of the three biological replicates for the six studied varieties. Means ± SD of single independent experiments and Coefficient of Variation (CV) are reported.

		CiAct2									*CiTub-2*								
	Sample	Ct value	Experiment mean	SD	Experiment Total Mean	SD	CV	Total Mean	SD	CV	Ct Value	Experiment mean	SD	Experiment Total Mean	SD	CV	Total Mean	SD	CV
Selvatica	Shoot CTRL1	24.83	25.0866667	0.2708858	24.7083333	0.48730348	**0.01881843**	24.5705556	0.651901	**0.026522**	28.16	28.4233333	0.51781248	28.52	0.38288579	**0.01443755**	28.1570278	1.38475158	**0.05293994**
	Shoot CTRL2	25.06									27.02								
	Shoot CTRL3	25.37									28.09								
	Drought stress1	24.46	24.33	0.2066065							28.7	28.6166667	0.26501572						
	Drought stress2	24.09									28.33								
	Drought stress3	24.44									28.83								
Zuccherina	Shoot CTRL1	24.78	25.0533333	0.2386071	24.975	0.48905837	**0.01633858**				25.807	28.089	0.40508858	27.1245	1.24663744	**0.04585112**			
	Shoot CTRL2	25.56									28.64								
	Shoot CTRL3	25.22									25.76								
	Drought stress1	24.33	24.8966667	0.583665							28.317	28.16	0.40560221						
	Drought stress2	24.59									28.569								
	Drought stress3	25.57									27.63								
Brindisina	Shoot CTRL1	24.1	24.49	0.4687217	24.8866667	0.62044782	**0.02525238**				28.74	26.8066667	0.140118	27.006	0.01834287	**0.00282517**			
	Shoot CTRL2	24.36									28.94								
	Shoot CTRL3	25.01									27.01								
	Drought stress1	25.90	25.2833333	0.5437217							27.93	27.2753333	0.91019876						
	Drought stress2	24.78									28.238								
	Drought stress3	25.21									27.66								
Esportazione	Shoot CTRL1	24.04	23.9866667	0.283784	24.6733333	0.77042714	**0.03158966**				25.718	24.865	0.73858279	26.033	**1.42026495**	**0.05455622**			
	Shoot CTRL2	23.68									24.38								
	Shoot CTRL3	24.24									24.5								
	Drought stress1	25.19	25.36	0.153948							28.473	27.201	0.63617057						
	Drought stress2	25.4									27.65								
	Drought stress3	25.49									27.48								
Rossa	Shoot CTRL1	24.04	23.5133333	0.4742714	23.995	0.71098526	**0.0300048**				23.778	23.9866667	0.16677884	24.4283333	0.5852612	**0.02396616**			
	Shoot CTRL2	23.12									24.087								
	Shoot CTRL3	23.36									24.1								
	Drought stress1	24.25	24.4766667	0.612318							25.31	24.85	0.51730088						
	Drought stress2	24.01									24.89								
	Drought stress3	25.17									24.29								
Galatina	Shoot CTRL1	24.14	23.89	0.25	24.185	0.37324255	**0.01543281**				24.37	24.2233333	0.18565146	25.7583333	1.6868333	**0.06560336**			
	Shoot CTRL2	23.89									24.17								
	Shoot CTRL3	23.64									24.43								
	Drought stress1	24.45	24.48	0.1571623							27.25	27.2833333	0.18878794						
	Drought stress2	24.34									27.13								
	Drought stress3	24.65									27.5								

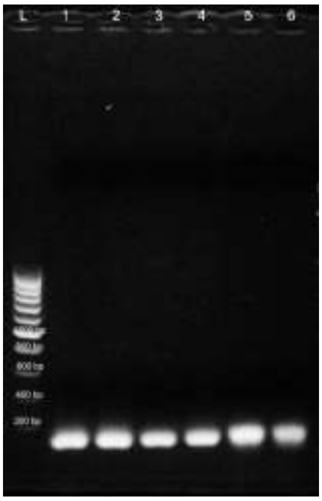

Figure S4. 2.0% agarose gel showing *CiXTH29* amplicon sizes for the different variety. Lane L: 200bp DNA ladder, *CiXTH29* PCR amplimer (~ 100 bp) obtained from Selvatica (lane 1), Zuccherina di Trieste (lane 2), Brindisina (lane 3), Esportazione (lane 4), Rossa Italiana (lane 5) and Galatina (lane 6) variety cDNA.

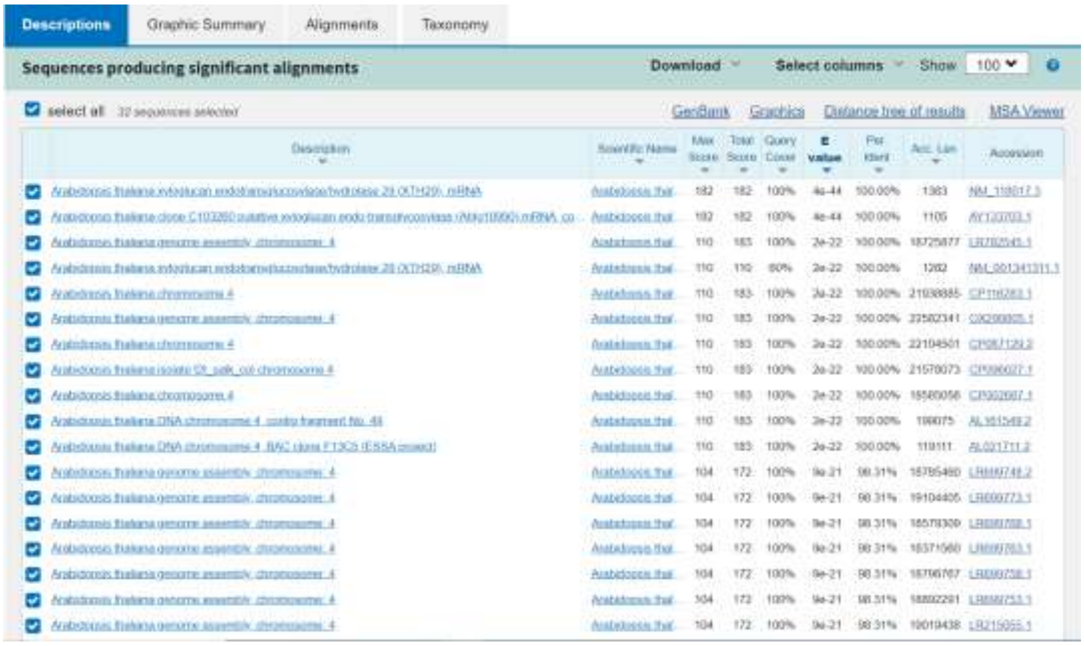

Description	Scientific Name	Max Score	Total Score	Query Cover	E value	Per. Ident	Acc. Len	Accession
Arabidopsis thaliana xyloglucan endotransglucosylase/hydrolase 29 (XTH29), mRNA	Arabidopsis thal...	182	182	100%	4e-44	100.00%	1383	NM_118017.3
Arabidopsis thaliana clone C103260 putative xyloglucan endo-transglycosylase (At4g18990) mRNA, co...	Arabidopsis thal...	182	182	100%	4e-44	100.00%	1105	AY133703.1
Arabidopsis thaliana genome assembly, chromosome: 4	Arabidopsis thal...	110	183	100%	2e-22	100.00%	18725877	LR782545.1
Arabidopsis thaliana xyloglucan endotransglucosylase/hydrolase 29 (XTH29), mRNA	Arabidopsis thal...	110	110	80%	2e-22	100.00%	1262	NM_001341311.1
Arabidopsis thaliana chromosome 4	Arabidopsis thal...	110	183	100%	2e-22	100.00%	21938885	CP116283.1
Arabidopsis thaliana genome assembly, chromosome: 4	Arabidopsis thal...	110	183	100%	2e-22	100.00%	23582341	[illegible]
Arabidopsis thaliana chromosome 4	Arabidopsis thal...	110	183	100%	2e-22	100.00%	22104501	CP087129.2
Arabidopsis thaliana isolate Of_salk_col chromosome 4	Arabidopsis thal...	110	183	100%	2e-22	100.00%	21578073	CP096027.1
Arabidopsis thaliana chromosome 4	Arabidopsis thal...	110	183	100%	2e-22	100.00%	18585056	CP002687.1
Arabidopsis thaliana DNA chromosome 4, contig fragment No. 48	Arabidopsis thal...	110	183	100%	2e-22	100.00%	199075	AL161549.2
Arabidopsis thaliana DNA chromosome 4, BAC clone F13C5 (ESSA project)	Arabidopsis thal...	110	183	100%	2e-22	100.00%	119111	AL021711.2
Arabidopsis thaliana genome assembly, chromosome: 4	Arabidopsis thal...	104	172	100%	9e-21	98.31%	18785480	[illegible]
Arabidopsis thaliana genome assembly, chromosome: 4	Arabidopsis thal...	104	172	100%	9e-21	98.31%	19104405	[illegible]
Arabidopsis thaliana genome assembly, chromosome: 4	Arabidopsis thal...	104	172	100%	9e-21	98.31%	18579309	[illegible]
Arabidopsis thaliana genome assembly, chromosome: 4	Arabidopsis thal...	104	172	100%	9e-21	98.31%	18371560	[illegible]
Arabidopsis thaliana genome assembly, chromosome: 4	Arabidopsis thal...	104	172	100%	9e-21	98.31%	18796767	[illegible]
Arabidopsis thaliana genome assembly, chromosome: 4	Arabidopsis thal...	104	172	100%	9e-21	98.31%	18892291	[illegible]
Arabidopsis thaliana genome assembly, chromosome: 4	Arabidopsis thal...	104	172	100%	9e-21	98.31%	19019438	[illegible]

Figure S5. Comparison of *CiXTH29* amplimer nucleotide sequence with *AtXTH29* nucleotide sequence.

		mRNA amount	
		CiXTH29	
		Mock	Drought
		$2^{-\Delta Cq}$ ± SD	$2^{-\Delta Cq}$ ± SD
	**Selvatica**	0,00340 ± 0,00040	0,0023± 0,00020
	**Zuccherina**	0,00270 ± 0,000040	0,0038 ± 0,00070
	**Brindisina**	0,00051 ± 0,00009	0,00426 ± 0,00037
	**Esportazione**	0,00098 ± 0,00060	0,00532 ± 0,00033
	**Rossa**	0,00111 ± 0,00021	0,005322 ± 0,00067
	**Galatina**	0,00147 ± 0,00038	0,00716 ± 0,00067

Figure S6. Amplification output values of *CiXTH29* mRNAs in mock condition and after drought stress. The values are expressed as $2^{-\Delta Cq}$ ± SD and are considered as proportional to the amount of mRNA target according to [56].

		mRNA amount	
		CiLEA4	
		Mock	Drought
		$2^{-\Delta Cq}$ ± SD	$2^{-\Delta Cq}$ ± SD
	Selvatica	0,00390 ± 0,00113	0,00450± 0,00063
	Zuccherina	0,00171 ± 0,00038	0,00137 ± 0,00029
	Brindisina	0,00083 ± 0,00030	0,00475 ± 0,00123
	Esportazione	0,00075 ± 0,00012	0,00337 ± 0,00027
	Rossa	0,00047 ± 0,00005	0,00199 ± 0,00014
	Galatina	0,00096 ± 0,00015	0,00228 ± 0,00017

Figure S7. Amplification output values of *CiLEA4* mRNAs in mock condition and after drought stress. The values are expressed as $2^{-\Delta Cq}$ ± SD and are considered as proportional to the amount of mRNA target according to [56].
